# How to improve image quality of DWI of the prostate—enema or catheter preparation?

**DOI:** 10.1007/s00330-021-07842-9

**Published:** 2021-03-23

**Authors:** Carolin Reischauer, Timmy Cancelli, Sonaz Malekzadeh, Johannes M. Froehlich, Harriet C. Thoeny

**Affiliations:** 1 Department of Medicine, University of Fribourg, Chemin du Musée 8, 1700 Fribourg, CH Switzerland; 2 Department of Radiology, Cantonal Hospital Fribourg, Fribourg, Switzerland

**Keywords:** Prostate, Diffusion magnetic resonance imaging, Artifacts, Enema, Suction

## Abstract

**Objectives:**

To compare the impact of laxative enema preparation versus air/gas suction through a small catheter on image quality of prostate DWI.

**Methods:**

In this single-center study, 200 consecutive patients (100 in each arm) with either enema or catheter preparation were retrospectively included. Two blinded readers independently assessed aspects of image quality on 5-point Likert scales. Scores were compared between groups and the influence of confounding factors evaluated using multivariable logistic regression. Prostate diameters were compared on DWI and T_2_-weighted imaging using intraclass correlation coefficients.

**Results:**

Image quality was significantly higher in the enema group regarding the severity of susceptibility-related artifacts (reader 1: 0.34 ± 0.77 vs. 1.73 ± 1.34, reader 2: 0.38 ± 0.86 vs. 1.76 ± 1.39), the differentiability of the anatomy (reader 1: 3.36 ± 1.05 vs. 2.08 ± 1.31, reader 2: 3.37 ± 1.05 vs. 2.09 ± 1.35), and the overall image quality (reader 1: 3.66 ± 0.77 vs. 2.26 ± 1.33, Reader 2: 3.59 ± 0.87 vs. 2.23 ± 1.38) with almost perfect inter-observer agreement (*κ* = 0.92–0.95). In the enema group, rectal distention was significantly lower and strongly correlated with the severity of artifacts (reader 1: *ρ* = 0.79, reader 2: *ρ* = 0.73). Furthermore, there were significantly fewer substantial image distortions, with odds ratios of 0.051 and 0.084 for the two readers which coincided with a higher agreement of the prostate diameters in the phase-encoding direction (0.96 vs. 0.89).

**Conclusions:**

Enema preparation is superior to catheter preparation and yields substantial improvements in image quality.

**Key Points:**

*• Enema preparation is superior to decompression of the rectum using air/gas suction through a small catheter.*

*• Enema preparation markedly improves the image quality of prostate DWI regarding the severity of susceptibility-related artifacts, the differentiability of the anatomy, and the overall image quality and considerably reduces substantial artifacts that may impair a reliable diagnosis.*

## Introduction

Multi-parametric MRI (mpMRI) of the prostate is increasingly gaining importance for the diagnosis and staging of prostate cancer. In order to achieve a greater level of standardization and consistency in the interpretation of mpMRI, the Prostate Imaging - Reporting and Data System (PI-RADS) has been developed [1–3]. Among the utilized sequences, DWI is the primary determining sequence for the diagnosis of prostate cancer in the peripheral zone where the majority of cancers (70–75 %) occur [2]. Beyond that, the addition of DWI helps considerably in discerning malignant nodules in the transition zone [2].

DWI is typically acquired using single-shot echo planar imaging due to its high signal-to-noise ratio (SNR) efficiency and its insensitivity to motion compared to conventional multi-shot techniques but the sequence is prone to image distortions and/or signal dropout caused by local susceptibility variations due to the presence of air and/or stool in the rectum in close proximity to the peripheral zone [4, 5]. Prostate MRI has been well established at both 1.5 T and 3 T but due to its intrinsically low SNR, DWI benefits from the transition to higher field strengths and the associated SNR gain but at the same time susceptibility-induced artifacts are amplified. It has been shown that these artifacts may be mitigated by the application of reduced field-of-view (FOV) approaches such as inner-volume excitation but residual image distortions may still be observed [6–10].

The PI-RADS Steering Committee has suggested patient preparation strategies that may improve the image quality of DWI but has been unable to reach a consensus on their value. The administration of a preparatory enema in the hours prior to the exam may reduce susceptibility-related artifacts. At the same time, enema preparation may promote peristalsis, amplifying motion-related artifacts [2]. Alternatively, the rectum may be decompressed using air/gas suction through a small catheter [2]. Several studies have investigated the impact of preparatory enemas on image quality of DWI but with conflicting results [11–13]. In addition, enema preparation has not been compared with other methods of patient preparation. Thus, further studies are needed to evaluate the value of enema preparation, in particular relative to other methods.

Therefore, the aim of the present retrospective study was to assess image quality of DWI in patients with laxative enema preparation compared with patients after decompression of the rectum using air/gas suction through a small catheter in a cohort of 200 consecutive patients who underwent mpMRI at 3 T at our institution including DWI with inner-volume excitation.

## Materials and methods

### Patient population and patient preparation

This retrospective, single-center study was approved by the Cantonal Ethical Committee of Bern with BASEC number 2019-00826. Due to the retrospective nature of the study, informed consent was waived.

All consecutive patients undergoing mpMRI of the prostate at our institution between 1 January 2017 and 15 July 2019 were included. In an attempt to reduce the occurrence of susceptibly-related artifacts, cleansing enema preparation was introduced into the routine protocol in October 2018. Before this date, patients underwent mpMRI after a radiology technician had removed excess gas in the rectum (catheter preparation group). For this purpose, the patient was positioned on the MR table in decupitus lateralis. Thereafter, a small rectal catheter (closed end, with two lateral eyes, 40 cm long, 25 Ch, Dahlhausen Medical Equipment) connected to a syringe (Omnifix®, 50 ml, B. Braun Medical) was placed by a radiology technician to gently aspirate rectal air/gas immediately before the examination. Patients of the enema preparation group rectally self-administered a laxative cleansing enema (Freka-Clyss® 133 ml, Fresenius Kabi) approximately 15 min prior to the exam. To mitigate motion-induced artifacts, all patients were administered scopolamine butylbromide (Buscopan®, 20 mg, Sanofi-Aventis) intravenously immediately prior to the exam.

### MRI acquisition

All patients underwent mpMRI of the prostate at 3 T (Discovery MR750 3.0 T, GE Healthcare) in the supine position including T_1_-weighted imaging (Dixon), T_2_-weighted imaging, DWI, and DCE-MRI. To diminish susceptibility-related artifacts, axial DWI of the prostate was performed using a reduced FOV methodology with inner-volume excitation, FOV optimized and constrained undistorted single-shot (FOCUS, GE Healthcare) DWI. At the same time as the introduction of cleansing enema preparation into the clinical routine, the mpMRI protocol was adjusted to adhere to PI-RADS recommendations. In patients of the catheter group, FOCUS DWI was performed using only two *b* values while in patients of the enema group, multiple *b* values were acquired. The DWI sequence parameters of both patient groups (catheter versus enema group) are summarized in Table 1. All examinations were performed without endorectal coils for signal reception.

**Table 1:** Sequence parameters of FOCUS DWI in the patient groups

	Catheter preparation	Enema preparation
FOV (mm^2^)	240 × 120	220 × 110
Acquisition matrix	160 × 80	136 × 68
In-plane resolution (mm^2^)	1.5 × 1.5	1.6 × 1.6
Slice thickness/gap (mm)	4/0.4	3.5/0.2
Phase-encoding direction	Anterior-posterior	Anterior-posterior
TE (ms)	58.2	68.6
TR (ms)	3700	4500
Echo train length (ms)	41	34
*b* values (s/mm^2^)	50,1500	50, 100, 200, 900, 1300, 2000
Number of signal averages	4, 16	3, 3, 5, 12, 16, 17

### Image analysis

For qualitative assessment of image quality, MR images were reviewed independently by two board-certified radiologists (reader 1 (T.C.) = 4 years and reader 2 (S.M.) = 5 years of experience in pelvic MRI) on a PACS workstation (Centricity PACS, GE Healthcare, AW Server 3.2 Ext. 3.2). Anonymized cases were evaluated in random order and without knowledge of the applied type of patient preparation and of patient history. Prior to reviewing patient scans, both readers were trained using exemplary data (not included in the study cohort) to ensure consistent ratings.

Using 5-point Likert scales, qualitative assessment of the DWI data was performed jointly using the diffusion-weighted images and the ADC maps. In addition to the *b* value images with *b* = 50 s/mm^2^, the *b* value images with *b =* 1500 s/mm^2^ and *b* = 1300 s/mm^2^ were evaluated in the catheter and enema groups, respectively. First, severity of susceptibility-induced image artifacts was determined: 0 = no, 1 = minor, 2 = moderate, 3 = severe, 4 = extensive. Second, the differentiability of the anatomy (central gland, peripheral gland, and pseudocapsule demarcation) was assessed: 0 = no differentiation, 1 = poorly identifiable, 2 = blurry, 3 = good, 4 = excellent. Third, overall image quality was graded: 0 = non-diagnostic, 1 = poor, 2 = moderate, 3 = good, 4 = excellent [4, 7–9, 11]. For the assessment of image distortions, DWI and T_2_-weighted images were co-registered using a rigid transform.

The degree of rectal distention was evaluated on T_2_-weighted images: 0 = none, 1 = minimal, 2 = small, 3 = moderate, 4 = large [14] and rectal content was classified: 0 = collapsed rectum, 1 = air only (> 80 %), 2 = stool only (> 80 %), 3 = air and stool, 4 = liquid/fluid or mixture (> 80 %). T_2_-weighted images were evaluated for the presence or absence of blurring (based on sharpness of the prostatic pseudocapsule, neurovascular bundle, and seminal vesicles compared to the peri-prostatic fat) and motion-related artifacts (movement or ghosting) [4, 15].

The extent of distortion artifacts and the anatomic agreement of FOCUS DWI and T_2_-weighted imaging was assessed quantitatively [7]. To this end, the axial slice covering the largest extent of the prostate on T_2_-weighted imaging was selected. The maximum diameters of the prostate in right-left (RL) and anterior-posterior (AP) direction were determined by the second reader on this slice and on the corresponding slice of the *b* value image with *b* = 50 s/mm^2^ [7]. In addition, prostate volumes were determined according to PI-RADS v2.1 recommendations [3].

### Statistical analysis

The statistical analysis was conducted using the computing environment R (R Foundation for Statistical Computing, version 4.0.0) and RStudio (RStudio, PBC, version 1.2.5042). A *p* value of < 0.05 was assumed to indicate statistical significance.

Age, prostate volumes, and PSA levels were compared between patient groups using independent-samples *t* tests. PSA values and prostate volumes were log transformed prior to the analysis to meet the normality assumption. Ordinal scores and binary criteria were compared between patient groups using Wilcoxon rank sum tests and are reported as means ± standard deviation. Multivariate logistic regression was used to assess the influence of potential confounding factors (age, PSA levels, and prostate volumes). To this end, the three ordinal image quality scores were dichotomized: image artifacts scores of > 2 were considered substantial artifacts and it was assumed that differentiability and overall image quality scores of < 2 significantly impair a reliable diagnosis. Spearman’s rank correlation was used to evaluate the correlation of rectal distention with the ordinal scores assessing image quality of DWI. The level of inter-rater agreement was evaluated using Cohen’s kappa or where applicable using quadratic weighted Cohen’s kappa (0–0.2 = slight, 0.21–0.40 = fair, 0.41–0.60 = moderate, 0.61–0.80 = substantial, and 0.81–1.00 = almost perfect [16]).

Anatomic agreement of FOCUS DWI and T_2_-weighted imaging was assessed using intraclass correlation coefficients (ICCs, two-way random model). The model used the stricter absolute agreement option which measures the extent to which the same score was assigned.

## Results

### Patient population

A total of 253 patients were primarily included. Fifty-three patients were secondarily excluded due to previous treatment for prostate cancer (hormonal treatment, radiation therapy, or surgery) (*n* = 34), the presence of pelvic metalwork (*n* = 14), and missing FOCUS DWI and/or T_2_-weighted imaging (*n* = 5). The remaining 200 patients were included into the analysis, with the catheter and the enema groups each consisting of 100 patients. There was no significant difference in age (*p* = 0.25) and prostate volume (*p* = 0.80) but a significant, albeit small, difference in PSA values (*p* = 0.01). A summary of the patient characteristics and MRI indications can be found in Tables 2 and 3. In two patients of the enema group, scopolamine butylbromide was not administered due to the presence of glaucoma. Another two patients of the enema and one patient of the catheter group received glucagon (GlucaGen®, 1 mg, Novo Nordisk) instead of scopolamine butylbromide.

**Table 2:** Characteristics of the patient groups

	Catheter preparation	Enema preparation
Number of patients	100	100
Age (years)	69.0 (42–89)	67.7 (42–85)
Prostate volume (ml)	55.4 (17.0–289.2)	56.3 (12.4–158.2)
PSA values (ng/ml)	13.1 (0.0–145.4)	10.4 (0.4–59.6)

Continuous data are reported as means and ranges; back projections were performed where appropriate

*PSA* prostate specific antigen

**Table 3:** Indications for MRI in the patient groups

	Catheter preparation	Enema preparation
High PSA levels	53	65
Staging	33	17
Abnormal DRE	0	13
PCa follow-up	4	0
Family history of PCa	0	2
Active surveillance	2	1
Other	8	2

*PSA* prostate specific antigen, *DRE* digital rectal examination, *PCa* prostate cancer

### Assessment of image quality

**Fig. 1:**
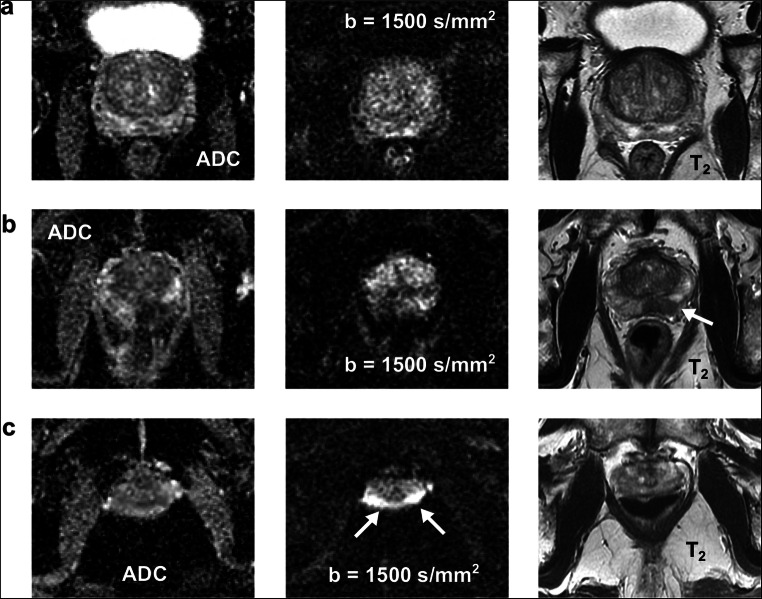
Exemplary ADC maps as well as diffusion-weighted and T_2_-weighted images of three patients with catheter preparation: **a** A 63-year-old patient with a collapsed rectum with excellent DWI quality regarding the severity of susceptibility-induced artifacts, the differentiability of the anatomy, and the overall image quality (both readers). **b** A 68-year-old patient with a moderate amount of air in the rectum which led to severe image distortions, poorly identifiable anatomy, and poor overall image quality (both readers); a tumor is visible in the peripheral zone on the T_2_-weighted image (see arrow) and may be suspected on the ADC image, but not on the high *b* value image. **c** A 78-year-old patient with a large amount of air which led to extensive image distortions (see arrows), non-diagnostic images as well as poorly identifiable (reader 1) and non-differentiable anatomy (reader 2); motion and blurring artifacts are present on the T_2_-weighted image (both readers)

**Fig. 2:**
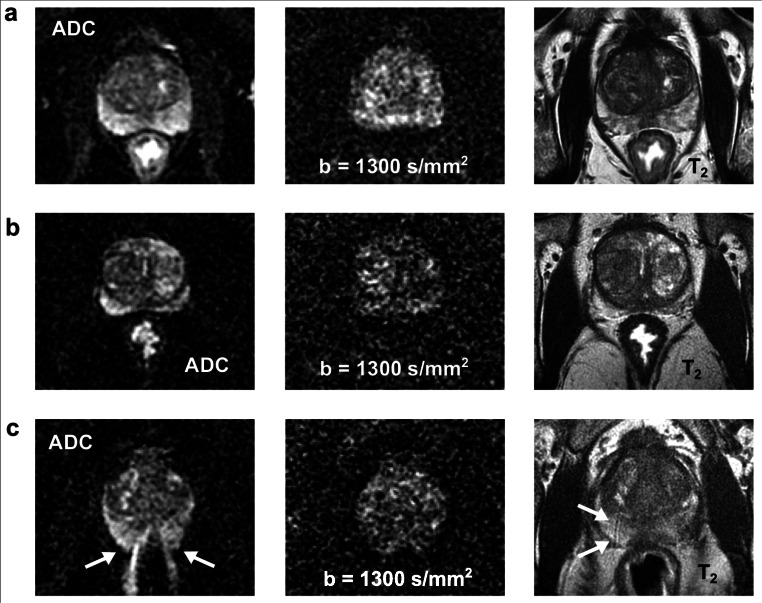
Exemplary ADC maps as well as diffusion-weighted and T_2_-weighted images of three patients with enema preparation: **a** A 77-year-old patient with a collapsed rectum and excellent DWI quality with respect to the severity of susceptibility-induced artifacts, the differentiability of the anatomy, and the overall image quality (both readers). **b** A 65-year-old patient with a small amount of liquid/fluid in the rectum but still excellent DWI quality (both readers). **c** A 59-year-old patient with a moderate amount of air in the rectum which led to severe image distortions (see arrows), poorly identifiable anatomy, and poor overall image quality; motion and blurring artifacts are visible on the T_2_-weighted image (see arrows, both readers)

Figures 1 and 2 show exemplary ADC maps as well as diffusion-weighted and T_2_-weighted images as well as corresponding image quality scores of patients with catheter and enema preparation.

**Fig. 3:**
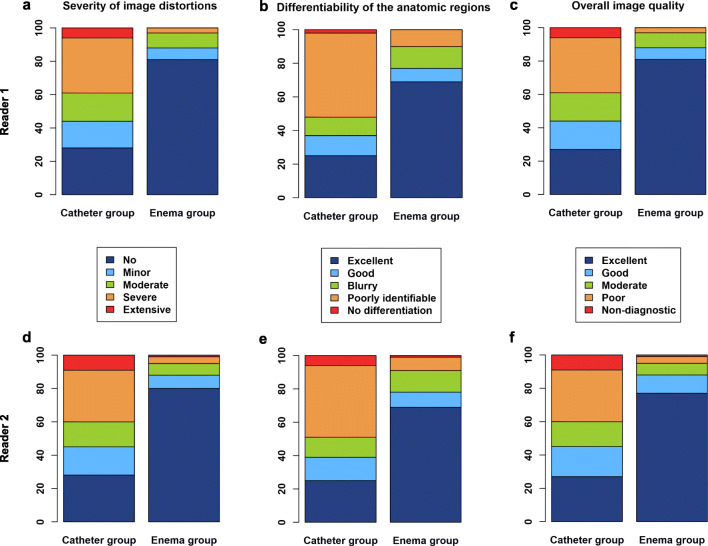
Assessment of the severity of image distortions (**a**, **d**), the differentiability of the anatomy (**b**, **e**), and the overall image quality (**c**, **f**) on 5-point Likert scales by the two readers (reader 1: first row, reader 2: second row) across the patient groups

Figure 3 summarizes the results of the assessment of image quality. Susceptibility-induced image distortions were significantly less severe (*p* < 0.001 for both readers) in patients with enema preparation (reader 1: 0.34 ± 0.77, reader 2: 0.38 ± 0.86) compared with patients undergoing catheter preparation (reader 1: 1.73 ± 1.34, reader 2: 1.76 ± 1.39). Similarly, differentiability of the anatomy was rated significantly higher (*p* < 0.001 for both readers) in the enema group (reader 1: 3.36 ± 1.05, reader 2: 3.37 ± 1.05) compared with the catheter group (reader 1: 2.08 ± 1.31, reader 2: 2.09 ± 1.35). Likewise, overall image quality was significantly higher (*p* < 0.001 for both readers) in the enema group (reader 1: 3.66 ± 0.77, reader 2: 3.59 ± 0.87) compared with the catheter group (reader 1: 2.26 ± 1.33, reader 2: 2.23 ± 1.38).

The level of inter-rater agreement was almost perfect for the ratings of artifact severity (catheter group: *κ* = 0.95, enema group: *κ* = 0.92), differentiability of the anatomy (catheter group: *κ* = 0.95, enema group: *κ* = 0.92), and overall image quality (catheter group: *κ* = 0.94, enema group: *κ* = 0.92).

For both readers, neither age, prostate volume, nor PSA values were significant predictors for either of the three ordinal image quality scores according to multivariable logistic regression (*p* < 0.05). Patients undergoing enema preparation showed significantly fewer substantial artifacts (*p* < 0.001 for both readers) with odds ratios of 0.051 (95 % CI: 0.012, 0.151) and 0.084 (95 % CI: 0.027, 0.208) for readers 1 and 2. Similarly, patients in the enema group showed significantly less critically low differentiability of the anatomy (*p* < 0.001 for both readers) with odds ratios of 0.109 (95 % CI: 0.048, 0.227) and 0.110 (95% CI: 0.047, 0.236). Likewise, patients in the enema group featured significantly less critically low overall image quality that may impede a reliable diagnosis (*p* < 0.001 for both readers) with odds ratios of 0.051 (95 % CI: 0.012, 0.151) and 0.084 (95 % CI: 0.027, 0.208).

### Assessment of rectal distention and content

**Fig. 4:**
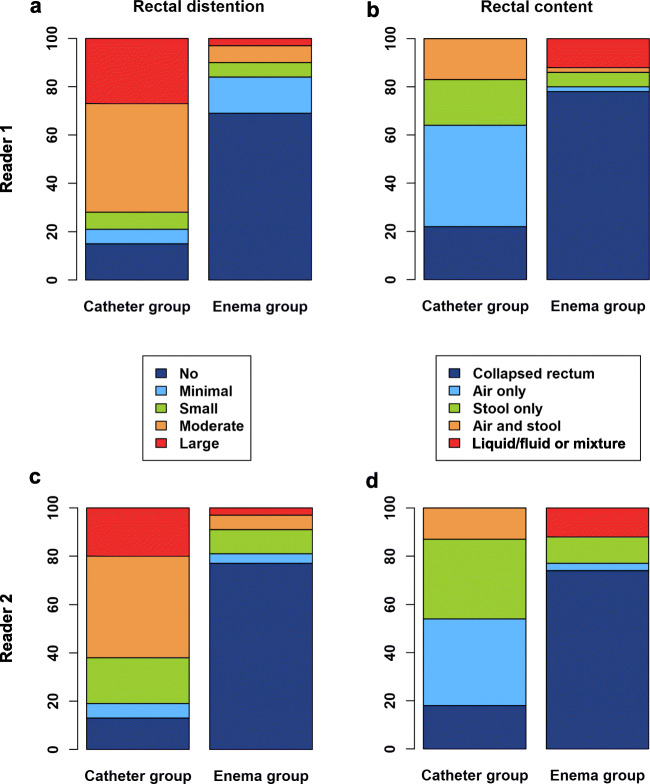
Assessment of the degree of rectal distention (**a**, **c**) on a 5-point Likert scale and classification of rectal content (**b**, **d**) by the two readers (reader 1: first row, reader 2: second row)

Figure 4 summarizes the results of the assessment of rectal distention and rectal content. The degree of rectal distention was significantly lower (*p* < 0.001 for both readers) in the enema group (reader 1: 0.60 ± 1.07, reader 2: 0.54 ± 1.09) than in the catheter group (reader 1: 2.65 ± 1.35, reader 2: 2.50 ± 1.25). Assessment of the rectal content revealed that more than one-third of the patients in the catheter group featured an “air only”-filled rectum while the great majority of patients in the enema group exhibited a “collapsed rectum.” As a result of the laxative enema, 12 patients of the enema group exhibited “liquid/fluid or mixture” in the rectum (both readers, see Fig. 4 b, d).

The level of inter-rater agreement was almost perfect for the assessment of rectal distention (catheter group: *κ* = 0.88, enema group: 0.91), substantial for the assessment of rectal content in the enema group (*κ* = 0.80), and moderate for the assessment of rectal content in the catheter group (*κ* = 0.56).

### Correlation of image quality scores and rectal distention

There was a strong positive correlation of the severity of susceptibility-related image distortions (reader 1: *p* < 0.001, *ρ* = 0.79; reader 2: *p* < 0.001, *ρ* = 0.73) and a strong negative correlation of the differentiability of the anatomy (reader 1: *p* < 0.001, *ρ* = - 0.68; reader 2: *p* < 0.001, *ρ* = - 0.63) and the overall image quality (reader 1: *p* < 0.001, *ρ* = -0.79; reader 2: *p* < 0.001, *ρ* = - 0.72) with the degree of rectal distention.

### Assessment of motion and blurring on T_2_-weighted imaging

There was no significant difference in the presence of blurring on T_2_-weighted images between groups (reader 1: *p* = 0.158, reader 2: 0.089). Contrary, there were significantly fewer motion-induced artifacts present in patients with enema relative to catheter preparation (reader 1: *p* = 0.048, reader 2: *p* < 0.001).

The level of inter-rater agreement was moderate for both the assessment of blurring (catheter group: *κ* = 0.49, enema group: *κ* = 0.55) and of motion-related artifacts (catheter group: *κ* = 0.65, enema group: *κ* = 0.61).

### Quantitative assessment of anatomic agreement

For patients with enema preparation, there was a high anatomic agreement of DWI and T_2_-weighted imaging in both the RL and the AP direction as measured by ICCs (RL: 0.94 (95 % CI: 0.91, 0.96), AP: 0.96 (95 % CI: 0.91, 0.95)). In contrast, the anatomic agreement was lower in patients undergoing catheter preparation in the AP direction which corresponds to the phase-encoding direction of the DWI sequence than in the RL direction (RL direction: 0.98 (95 % CI: 0.95, 0.98), AP direction: 0.89 (95 % CI: 0.85, 0.93)).

### Discussion

Multi-parametric MRI of the prostate is increasingly being performed without the use of endorectal coils to increase patient comfort and to reduce costs and the duration of the examination. In this case, optimal patient preparation is of particular importance, since the presence of air and/or stool in the rectum in close proximity to the prostate may induce image distortions that degrade the image quality of DWI. No consensus has yet been reached concerning patient preparation for mpMRI of the prostate. In the present study, two methods of patient preparation were directly compared. The results show that the image quality of DWI was significantly higher in patients with enema relative to catheter preparation regarding the severity of susceptibility-related image artifacts, the differentiability of the anatomy, and the overall image quality. In particular, fewer substantial susceptibility-related artifacts were present in the enema group. The difference in image quality may be attributed to the lower degree of rectal distention in the enema group. Indeed, the great majority of patients with enema preparation exhibited a “collapsed rectum” while more than one-third of the patients undergoing catheter preparation featured an “air-only” filled rectum.

The patients generally tolerated both preparation methods well but it is likely that enema preparation features increased patient comfort since it may be self-administered whereas catheter preparation is performed by a radiology technician, which also increases the duration of examination and in turn costs. However, elderly patients may have difficulty self-administering an enema and administration of the enema by a radiology technician might be preferable in this case. Furthermore, several questions regarding enema preparation may require further clarification such as whether application of a single dose is sufficient and how the patient’s position during the administration impacts its effectiveness (standing position versus lateral decupitus).

Previous studies have evaluated the impact of enema preparation on image quality of DWI but with conflicting results [11–13]. Lim et al [11] found neither a significant improvement in image quality nor a significant reduction in image distortions and the severity of distortions did not correlate with the amount of rectal gas. These contrary findings may be explained by the fact that the relatively small study cohort of 60 patients contained no subjects with moderate or large amounts of gas in the rectum which would lead to pronounced artifacts. More recently, Coskun et al [12] concluded that enema preparation may diminish rectal gas but with minor effects on DWI distortions and overall image quality. In this study, patients self-administered a preparatory enema approximately 12 h prior to the exam (compared with approximately 15 min in the present work) which may have reduced its impact on image quality. Beyond that, there was only weak agreement between the two independent readers regarding the assessment of distortions and artifacts. In contrast, inter-rater agreement in the present study was almost perfect for the ordinal image quality scores, likely due to the fact that both readers were trained prior to reviewing patient scans. In agreement with our work, Plodeck et al [13] found that the application of an enema shortly before the exam significantly reduces both the incidence and severity of susceptibility-induced image artifacts but did not compare the results with alternative methods of patient preparation. In our study, two methods of patient preparation were comprehensively compared in a large patient cohort of 200 consecutive patients.

There have been concerns that enema preparation may promote peristalsis which may induce motion-related artifacts [2]. In the present study, fewer motion-induced artifacts were observed in patients with enema relative to catheter preparation which may be explained by the higher percentage of patients with a collapsed rectum. In principle, anorectal sensoric stimulation by either a catheter or an enema applicator may initially increase the colonic motility [17] while in the further course of time motility may decrease due to a collapsed rectum after effective laxation. However, there is an ongoing debate about whether visceral sensoric or rather biomechanical motoric effects dominate peristalsis [18]. In addition, intravenous administration of scopolamine butylbromide may have mitigated motion-related artifacts although previous work in patients without enema preparation showed no significant effect on image quality [19]. Further studies should evaluate whether the administration of an anti-peristaltic drug may be omitted.

There are several limitations to our work. First, the present work was a retrospective study. Second, there was a significant difference in PSA values between groups likely due to the fact that the overall indication to perform mpMRI of the prostate before biopsy has increased over time. However, according to multivariable logistic regression, PSA values were not significant predictors for either of the ordinal image quality scores. Third, although readers were blinded to the type of patient preparation, they may have inferred it from suggestive imaging findings such as fluid in the rectum. Fourth, there were differences in the parameters of the FOCUS DWI sequences between groups which could lead to varying sensitivities to susceptibility-induced artifacts. From a theoretical point of view, the sensitivity of an echo planar imaging sequence to image distortions is proportional to the echo train length and the scan resolution in phase-encoding direction [6]. Based on the sequence parameters given in Table 1, it may be deduced that the DWI sequence that was utilized in the catheter group shows a 1.12-times higher sensitivity to susceptibility-related image distortions. This inherently higher sensitivity to susceptibility-induced artifacts may have affected the results, but it cannot explain the much lower image quality that was seen in the catheter relative to the enema group. In particular, it cannot explain the much higher likelihood of observing substantial artifacts in the catheter group. Beyond that, the results regarding the incidence and severity of susceptibility-related image artifacts after enema application are in agreement with previous work [13]. Fifth, the image quality of DWI was assessed using subjective ordinal scales. However, inter-rater agreement was high and the approach was complemented by a quantitative analysis. Finally, while image quality was evaluated, impact on diagnostic accuracy was not assessed but it is likely that improved image quality will translate into a more reliable diagnosis.

In conclusion, the present study demonstrates that enema preparation immediately before the MRI exam is superior to catheter preparation regarding image quality of DWI and considerably reduces the occurrence of substantial susceptibility-induced image artifacts that may impede image interpretation and consequently correct diagnosis.

## Abbreviations

AP: Anterior-posterior
CI: Confidence interval
FOCUS: Field of view optimized and constrained undistorted single-shot
FOV: Field of view
ICC: Intraclass correlation coefficient
mpMRI: Multi-parametric MRI
PI-RADS: Prostate Imaging - Reporting and Data System
PSA: Prostate-specific antigen
RL: Right-left
SNR: Signal-to-noise ratio

## Declarations

### Guarantor

The scientific guarantor of this publication is Carolin Reischauer.

### Statistics and biometry

No complex statistical methods were necessary for this paper.

### Methodology

• retrospective

• observational

• performed at one institution
